# An anthropometric approach to characterising neonatal morbidity and body composition, using air displacement plethysmography as a criterion method

**DOI:** 10.1371/journal.pone.0195193

**Published:** 2018-03-30

**Authors:** Jacqueline Huvanandana, Angela E. Carberry, Robin M. Turner, Emily J. Bek, Camille H. Raynes-Greenow, Alistair L. McEwan, Heather E. Jeffery

**Affiliations:** 1 School of Electrical and Information Engineering, University of Sydney, Sydney, Australia; 2 School of Public Health and Community Medicine, University of New South Wales, Sydney, Australia; 3 Sydney Medical School, University of Sydney, Sydney, Australia; 4 Sydney School of Public Health, University of Sydney, Sydney, Australia; Liverpool John Moores University, UNITED KINGDOM

## Abstract

**Background:**

With the greatest burden of infant undernutrition and morbidity in low and middle income countries (LMICs), there is a need for suitable approaches to monitor infants in a simple, low-cost and effective manner. Anthropometry continues to play a major role in characterising growth and nutritional status.

**Methods:**

We developed a range of models to aid in identifying neonates at risk of malnutrition. We first adopted a logistic regression approach to screen for a composite neonatal morbidity, low and high body fat (BF%) infants. We then developed linear regression models for the estimation of neonatal fat mass as an assessment of body composition and nutritional status.

**Results:**

We fitted logistic regression models combining up to four anthropometric variables to predict composite morbidity and low and high BF% neonates. The greatest area under receiver-operator characteristic curves (AUC with 95% confidence intervals (CI)) for identifying composite morbidity was 0.740 (0.63, 0.85), resulting from the combination of birthweight, length, chest and mid-thigh circumferences. The AUCs (95% CI) for identifying low and high BF% were 0.827 (0.78, 0.88) and 0.834 (0.79, 0.88), respectively.

For identifying composite morbidity, BF% as measured via air displacement plethysmography showed strong predictive ability (AUC 0.786 (0.70, 0.88)), while birthweight percentiles had a lower AUC (0.695 (0.57, 0.82)). Birthweight percentiles could also identify low and high BF% neonates with AUCs of 0.792 (0.74, 0.85) and 0.834 (0.79, 0.88). We applied a sex-specific approach to anthropometric estimation of neonatal fat mass, demonstrating the influence of the testing sample size on the final model performance.

**Conclusions:**

These models display potential for further development and evaluation in LMICs to detect infants in need of further nutritional management, especially where traditional methods of risk management such as birthweight for gestational age percentiles may be variable or non-existent, or unable to detect appropriately grown, low fat newborns.

## Introduction

Neonatal body composition assessment plays an important role in characterising the nutritional and dietary status of newborn infants. Those with limited body fat face risks of increased mortality and morbidity, with undernutrition linked to inhibited long-term growth and cognitive development [1, 2]. A 2010 report from the World Health Organization (WHO) attributed undernutrition as a contributing factor in one third of child deaths under five years of age [3]. The majority of these deaths occur within the first few days of life and in low and middle income countries (LMICs) [4].

Current validated methods for measuring body composition such as air displacement plethysmography (ADP), dual x-ray absorptiometry and hydrometric methods are often impractical in LMICs, given stipulations of portability, cost and operational expertise. Anthropometric measures such as mid-upper arm circumferences (MUAC), birthweight for gestational age percentiles (henceforth birthweight percentiles) and weight-for-length Z scores are commonly used in place of these more complex techniques to gauge undernutrition [5].

Simple cut-offs have been defined for MUAC to screen for moderate and severe acute malnutrition and although they have been evaluated in older infants (aged 6–60 months) with respect to risk of mortality [6], there is a lack of similar data on its reliability and association with these risks in younger infants (under 6 months). MUAC and abdominal circumference also reflect adiposity [7]. Head circumference reflects brain volume and thus intrauterine brain development [8, 9] while chest circumference has been shown to be a significant predictor of low birthweight [10, 11], commonly used to identify infants at risk from undernutrition. Though these circumferences have not been extensively evaluated in relation to malnutrition risk, their simplicity and scalability may render them suitable candidates for screening use in LMICs. In the newborn period, birthweight percentiles and less often, weight-for-length Z scores are traditionally used to identify malnutrition, though as with all anthropometry, they may be susceptible to measurement inaccuracies. Birthweight percentiles are limited by unknown or inaccurate gestational age in LMIC settings and cannot detect the appropriately grown (10-90^th^ percentile) low fat newborn at risk of significant morbidity [12].

ADP has often been used as the reference method in infants, and has been previously validated for this population [13–15]. Carberry et al. have reported that body fat % (BF%) as measured by ADP offers a better composite measure of poor neonatal outcome than conventional birthweight measurements [12].

Anthropometric equations for the estimation of neonatal body fat have been developed against a range of reference methods. These include total body water as measured via total body electrical conductivity [16, 17], ADP [18, 19] and dual x-ray absorptiometry [20]. A recent validation of four anthropometric equations using skinfold thickness measurements demonstrated poor explanation of variance (R-squared ranging from 0.55–0.63) between the developed equations and against ADP [21]. High inter-individual variability in the first few days of life may have contributed to the poor agreement observed and there is thus a need for caution in interpretation of the results from predictive equations.

Most models for the estimation of neonatal body fat account for sex of the infants using a single variable in the linear regression model (often 1 = male, 0 = female) [16–19]. This may not allow sufficient adjustments for sex-specific anthropometry [22] and may be biased by the predominance of either sex in the dataset used for model development.

The aim of this work was to develop anthropometric models for various applications within the first few days post-delivery. We sought to develop logistic regression models for identifying infants at risk of malnutrition, first using a composite measure of neonatal morbidity previously developed [12], while the second and third were to screen for low and high BF% neonates measured via the reference method, ADP. We also developed a linear regression model using a sex-specific approach to directly estimate neonatal fat mass (FM) using anthropometric features and thus characterise nutritional status.

## Materials and methods

### Data collection

Eligible neonates were term (>37 weeks), singletons born at Royal Prince Alfred Hospital, Sydney during September and October 2010. Those with major congenital abnormalities were excluded from the study. Further details of recruitment and study data collection have been previously reported [12]. Briefly, there were 782 eligible neonates born during the study period, 581 of whom were enrolled in the study (75% recruitment rate). Of these, 524 neonates had valid and complete measurements and were included for model development.

Body composition data including BF% and FM was collected via ADP (PEA POD; COSMED, Concord, USA) and anthropometric measurements were collected within 48 hours of birth. ADP applies basic gas laws to determine the body volume from that of the air displaced by the infant in an enclosed chamber, maintained at a constant temperature. Together with the weight measurement from the PEA POD scales, the density of the subject can be determined and, assuming a two-compartment model and constant density for each fat and fat-free mass, the weights of each component can be determined. BF% measurements from ADP was used as the gold standard for subsequent model development.

Anthropometric measurements (length and head, mid-upper arm, mid-thigh, abdominal and chest circumferences) were standardised using skills-based educational methods and competency confirmation [23]. Length was measured to approximately 0.1 cm heel to crown using an Easy-Glide Bearing Infantometer (Perspective Enterprises, Portage, MI). Weight on day of measurement (henceforth, weight) was measured to the nearest gram using the integrated PEA POD scales. To simulate the accuracy of standard scales in LMICs, weight was subsequently rounded to the nearest 5 grams during pre-processing. Circumferences were measured using a paper tape measure. Anthropometry and length were taken by a single researcher except for a subset of approximately 40 infants where duplicate measurements were taken [24].

#### Ethics

The study was approved by the Human Research Ethics Committees of Royal Prince Alfred Hospital and the University of Sydney (HREC/09/RPAH645, SSA/09/RPAH646, and University of Sydney Ref. No. 12732). Informed parental written consent was obtained, and participation was voluntary.

### Model development and statistical analyses

Data processing and feature selection was completed in Python (Python Software Foundation, version 2.7.11 https://www.python.org/), with further statistical analysis undertaken in R 3.3.1 [25].

#### Neonatal morbidity screening

Composite neonatal morbidity was defined on the basis of hypothermia, poor feeding and extended length of stay, as previously described [12]. This composite measure associated with undernutrition was developed using univariate logistic regression to identify the combination of variables that could identify small-for-gestational age neonates based on birthweight percentiles [12]. We completed an exhaustive search of all possible combinations of linear, inverse and square transformations of anthropometric features. Note that for all measures, gestational age was excluded from the feature set for model development as this may be unknown or unreliable in LMICs. We also examined the greatest AUC achieved by a model excluding length as a feature and compared model performance using the Delong method [26], should length boards and appropriate training not be available.

Logistic regression models were constructed using a maximum of four original features, balancing computational efficiency and model performance. Receiver-operator characteristic (ROC) curves were generated for each feature combination, providing an indication of sensitivity and specificity in identifying the class denoting composite morbidity. The final models were selected based on those which maximised the area under the ROC curves (AUC), a measure of predictive ability.

#### Screening of low and high BF% neonates

A similar approach was adopted for screening of low and high BF% neonates, which were defined respectively as 1 SD below and above the mean, stratified by sex. These cut-offs were consistent with previous work finding the low BF% infants exhibited greater risk of composite neonatal morbidity [12]. Logistic regression models were developed independently using an exhaustive search for combinations of transformed features that yielded the greatest AUC for identifying low and high BF% neonates.

#### Estimation of neonatal fat

For the estimation of neonatal fat mass (FM) using a linear regression model, relevant features and commonly-used combinations of anthropometric measures were included in the complete set of features. We sought to characterise the underlying drivers behind weight-for-length ratio (W/L) and its higher powers, W/L^2^ (body mass index) and W/L^3^ (ponderal index) [27]. Inclusion of all three ratios would introduce multiple collinearity effects and thus, factor analysis was applied to determine the ratio for inclusion in the complete feature set. Weight rather than birthweight was used in these ratios given the weight loss observed in the first few postnatal days [28] and the varying ages at measurement.

To mitigate the influence of varying ranges, all continuous variables were standardised (mean = 0, SD = 1). Feature selection was then undertaken using recursive feature elimination, ranking features by their linear model coefficients, repeatedly removing them from the model and determining the optimal set of features. This was determined through minimisation of the root mean squared error score (RMSE) based on 10-fold cross-validation of the dataset. Once the set of features was determined, model fitting was completed on the sex-specific subgroups with the non-standardised features.

The performance of the sex-specific models was compared against combined sex models fitted to the determined feature set combined with a binary variable denoting sex (male = 1, female = 0). For the combined sex model, we also included sex-anthropometry interaction terms and examined the effect on the final model.

### Model evaluation

#### Logistic regression models

We compared the developed logistic regression models against common anthropometric indices using the Delong method for comparison of correlated ROC curves [26]. Logistic regression models were further evaluated using leave-one-out cross-validation, with models rejected if the AUC from this was less than or equal to 0.5. This involved using all samples except one in fitting the logistic regression model and subsequently evaluating the predicted probability of the omitted sample. The process was repeated for all samples and the probabilities used to construct a ROC curve for evaluation of the leave-one-out cross validation AUC.

#### Linear regression models for neonatal fat mass estimation

To investigate the motivation for sex-specific model fitting, we compared anthropometric and other characteristic differences between male and female neonates. We applied a student’s t-test for continuous variables such as birthweight, length and head circumference and a chi-squared test for categorical variables. We then compared the performance of sex-specific models for a range of test sample sizes by first dividing the dataset into sex-stratified halves, a training and a testing set. The male and female portions of training set were used to fit sex-specific linear estimation models while an equal-sized subset containing an even distribution of sexes was used to fit the combined sex model. We continually and randomly restricted the testing set, determining RMSE of fat-free mass, FM and BF% estimations for each sample size over 100 iterations. The overall process was repeated for 10 divisions of training and testing sets and the mean RMSE calculated for a given sample size.

## Results

The characteristics of the population are summarised in Table 1, with continuous variables expressed as mean and standard deviation (SD) and categorical variables as percentages (%).

**Table 1:** Comparisons of anthropometry and body composition measures for male and female neonates. An independent t-test (two-tailed) was applied to compare continuous variables and a chi-squared test for categorical variables (neonatal composite morbidity and proportions in each fat range). Statistical significance is denoted by *p<0.05, ***p<0.001.

Characteristics	Male	Female	p
n	272	252	
Birthweight (g)	3533 ± 475	3366 ± 411	<0.001***
Weight (g)	3359 ± 448	3193 ± 398	<0.001***
Length (cm)	50.4 ± 1.9	49.2 ± 1.7	<0.001***
Gestational age (weeks)	39.6 ± 1.1	39.5 ± 1.2	0.120
Age at measurement (days)	1.17 ± 0.6	1.19 ± 0.6	0.695
Mid-upper arm circumference (cm)	11.0 ± 1.0	10.9 ± 0.9	0.0897
Head circumference (cm)	35.0 ± 1.1	34.1 ± 1.1	<0.001***
Mid-thigh circumference (cm)	15.1 ± 1.3	15.0 ± 1.2	0.568
Abdominal circumference (cm)	30.6 ± 2.2	30.5 ± 2.0	0.511
Chest circumference (cm)	32.7 ± 1.8	32.4 ± 1.6	0.023*
Neonatal composite morbidity^a^ (%)	3.7	3.2	0.176
Proportion low fat (%)	12.5	14.7	0.100
Proportion moderate fat (%)	71.7	71	0.199
Proportion high fat (%)	15.8	14.3	0.143
Body fat %	8.89 ± 4.0	10.09 ± 3.9	<0.001***
Fat mass (g)	310 ± 167	332 ± 155	0.119

^a^Composite neonatal morbidity defined as a composite of hypothermia, poor feeding and extended length of stay. Previously described in [12]

### Logistic regression models

We developed logistic regression models to screen for a composite measure of neonatal morbidity as well as low and high BF% neonates.

#### Neonatal morbidity screening

**Fig 1:**
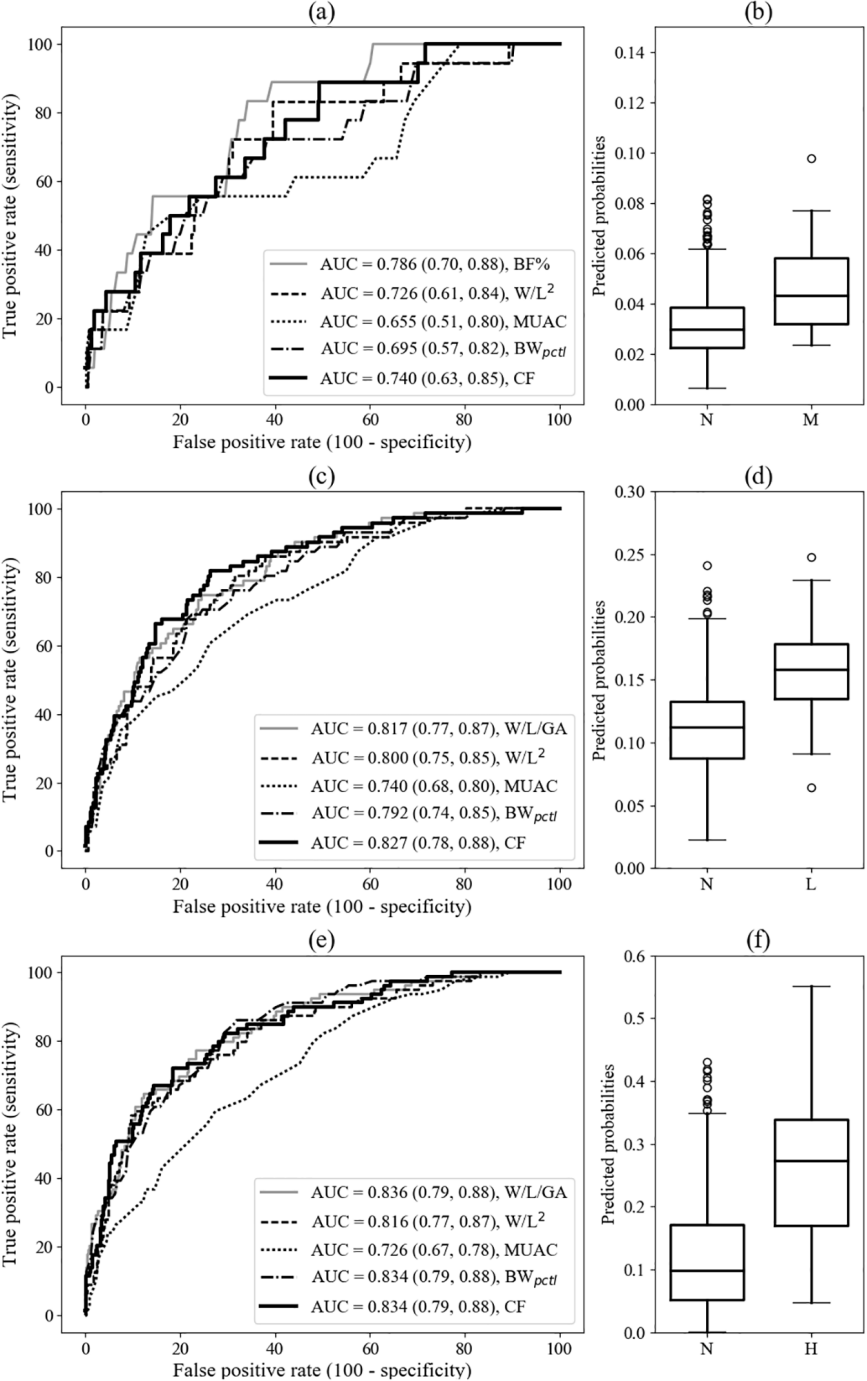
Receiver-operator characteristic curves and predicted probabilities for developed logistic regression models. Panels (a), (c) and (e) display the ROC curves for each of the developed (CF) models and other comparative models for the identification of composite neonatal morbidity [12], low BF% and high BF%, respectively. Comparative models include those fitted using body fat percentage (BF%), weight for length (W/L), mid-upper arm circumference (MUAC) and birthweight percentile (BW_pctl_). Corresponding boxplots in (b), (d) and (f) show the predicted probabilities from the corresponding CF logistic regression models for each of the two classes: negative (N) and positive (M: composite neonatal morbidity, L: low BF% and H: high BF%).

From the exhaustive search of all possible 4-feature models to screen for neonatal morbidity, the following features were frequently included in high scoring models: weight or birthweight, chest or abdominal circumference, mid-thigh circumference and length. The greatest AUC achieved was 0.740 (0.63, 0.85) by the combination of birthweight, length, chest and mid-thigh circumferences (Fig 1). The composite feature is defined in Eq 1, where circumference is denoted by *circ*.

$${CF}_{morbidity}=\frac{birthweight\times {chest}_{circ}}{length\times {thigh}_{circ}}$$ (1)

The greatest AUC for a model without length as a feature was 0.736 (0.62, 0.85) and combined the product of birthweight and abdominal circumferences, divided by that of head and mid-thigh circumferences. The AUC score difference between the reported model (Eq 1) and this length-free model was minimal and not significant.

We compared the ROC curves of the developed models with those of commonly used metrics. Table 2 reports the AUC, standard error and p-values from the Delong method for comparing correlated ROC curves [26]. The developed model (AUC 0.740 (0.63, 0.85)) displayed a high degree of overlap with the BF% ROC curve (AUC 0.786 (0.70, 0.88)) which exhibited the next highest AUC. MUAC had a significantly poorer AUC of 0.655 (0.51, 0.80) (p = 0.046) than that of BF%.

**Table 2:** Comparison of receiver-operator characteristic curves for the prediction of composite neonatal morbidity, low and high fat BF% using the Delong method [26]. For each pair of logistic regression models, the standard error and p-value from the Delong method for ROC curve comparison are reported [26]. Comparisons include BF% from ADP, weight-for-length-for-gestational age (W/L/GA), weight-for-length-squared (W/L^2^), mid-upper arm circumference (MUAC), birthweight percentiles (BW_pctl_) and developed composite feature (CF). Statistical significance is denoted by *p<0.05, **p<0.01 ***p<0.001.

Model	AUC (95% CI)	W/L^2^	MUAC	BW_pctl_	CF
**Composite neonatal morbidity**					
BF%	0.786 (0.70, 0.88)	0.055, 0.24	0.066, 0.046*	0.062, 0.141	0.061, 0.453
W/L^2^	0.726 (0.61, 0.84)		0.061, 0.239	0.047, 0.510	0.04, 0.729
MUAC	0.655 (0.51, 0.80)			0.067, 0.548	0.07, 0.227
BW_pctl_	0.695 (0.57, 0.82)				0.051, 0.376
CF	0.740 (0.63, 0.85)				
**Low BF%**					
W/L/GA	0.817 (0.77, 0.87)	0.012, 0.174	0.028, 0.006**	0.011, 0.031*	0.014, 0.455
W/L^2^	0.800 (0.75, 0.85)		0.029, 0.035*	0.021, 0.712	0.019, 0.141
MUAC	0.740 (0.68, 0.80)			0.030, 0.076	0.028, 0.002*
BW_pctl_	0.792 (0.74, 0.85)				0.018, 0.056
CF	0.827 (0.78, 0.88)				
**High BF%**					
W/L/GA	0.836 (0.79, 0.88)	0.011, 0.06	0.026, ***	0.010, 0.832	0.047, 0.961
W/L^2^	0.816 (0.77, 0.87)		0.028, 0.001**	0.177, 0.309	0.049, 0.715
MUAC	0.726 (0.67, 0.78)			0.026, ***	0.047, 0.02*
BW_pctl_	0.834 (0.79, 0.88)				0.044, 0.998
CF	0.834 (0.79, 0.88)				

#### Screening of low and high BF% neonates

The logistic regression models for low and high BF% exhibited AUCs of 0.827 (0.78, 0.88) and 0.834 (0.79, 0.88) respectively (Fig 1). The features used to construct the corresponding composite features are summarised in Eqs 2 and 3.

$${CF}_{low-fat}=\frac{{weight\;}^{2}}{length\times {chest}_{circ}}$$ (2)

$${CF}_{high-fat}=\frac{{head}_{circ}\times {length}^{2}}{birthweight\times weight}$$ (3)

### Linear regression models

#### Feature selection

Factor analysis was applied to the W/L ratio and its 2 higher powers to determine which of the ratios to be included in the set for subsequent feature selection and to avoid multiple collinearity effects. Results showed that 89.5% of the variance could be explained by a single underlying factor, driven mostly by W/L^2^ (R-squared > 0.99). This ratio exhibited a R-squared of 0.874 with W/L and 0.809 with W/L^3^. R-squared between W/L and W/L^3^ was 0.472.

Recursive feature elimination identified the combination of weight, head circumference, mid-thigh circumference and W/L^2^ as the optimal set of features for neonatal FM estimation.

#### Linear estimation of neonatal fat mass

The sex-specific and combined sex linear regression model coefficients are detailed in Table 3. Weight and W/L^2^ were significant predictors of male and female neonatal FM, whereas head circumference was significant (p = 0.018) in the male population only. All three models exhibited similar R-squared statistics of approximately 0.59. For the combined sex model, all variables including sex were significant predictors of neonatal fat mass. There were no significant interactions between sex and anthropometric features (p > 0.3).

**Table 3:** Linear regression model coefficients for estimation of neonatal fat mass in grams.

Variable	Intercept	Weight (g)	circ_head_ (cm)	circ_thigh_ (cm)	W/L^2^ (g/cm^2^)	Sex
**Male**						
Coefficient	-309.54	0.226	-19.93	15.74	243.53	-
SE	261.74	0.035	8.35	8.42	105.11	-
p	0.238	<0.001***	0.018*	0.063	0.021*	-
**Female**						
Coefficient	-677.90	0.190	-8.280	11.47	390.141	-
SE	270.16	0.038	8.705	7.95	105.060	-
p	0.013*	<0.001***	0.342	0.150	<0.001***	-
**Combined sex**						
Coefficient	-445.45	0.212	-14.857	13.191	312.795	-47.21
SE	186.35	0.0255	6.008	5.780	74.00	10.14
p	0.017*	<0.001***	0.014*	0.023*	<0.001***	<0.001***

R-squared statistics for the male, female and combined sex regression models were 0.589, 0.591 and 0.590, respectively. Units for each variable are shown in parentheses, with coefficients, standard error (SE) and p value from linear regression model fitting shown for the sex-specific and combined sex models. The variable denoting sex comprises 1 = male and 0 = female. Statistical significance is denoted by

*p<0.05,

***p<0.001.

#### Model evaluation

**Fig 2:**
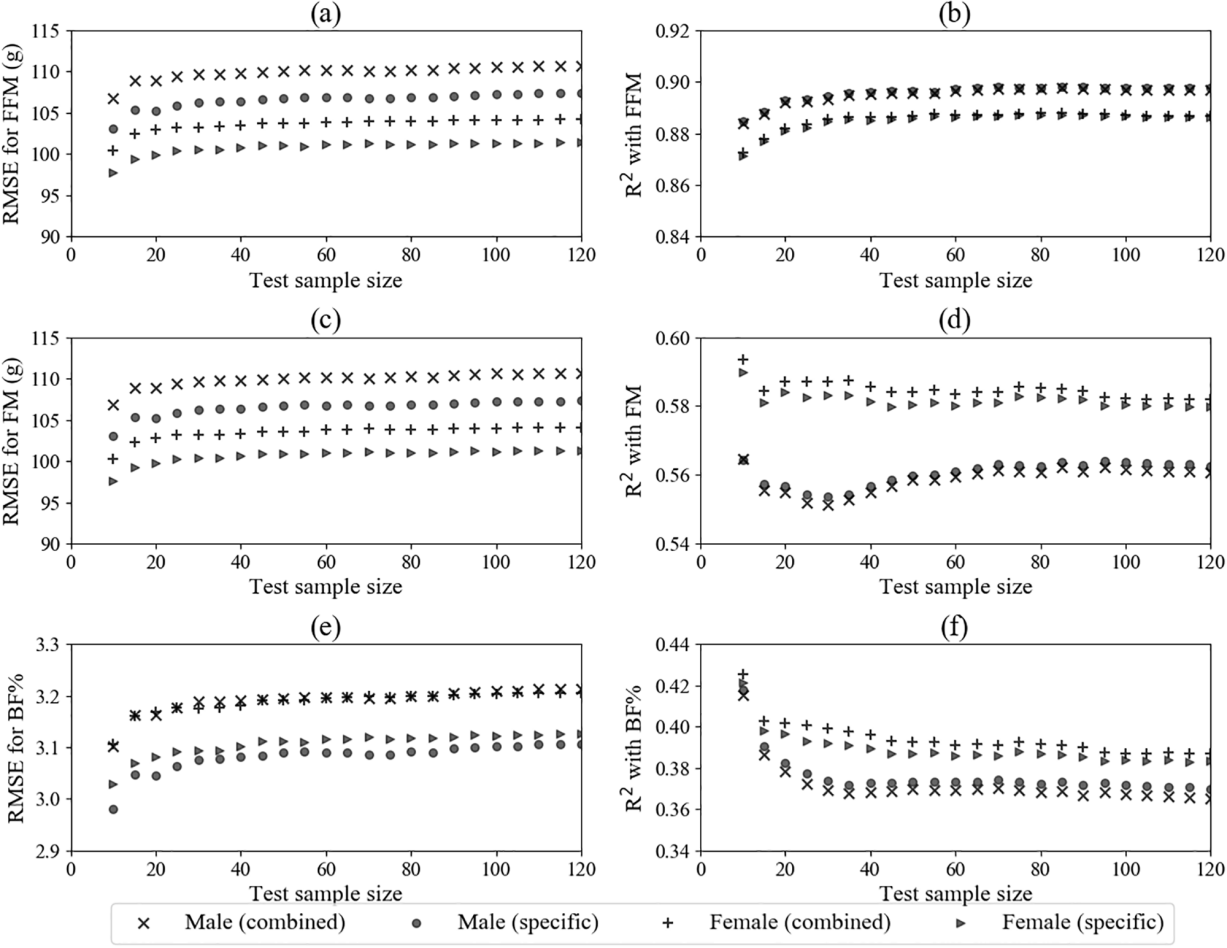
Mean model RMSE and R-squared statistics for estimations of body composition parameters at a testing sample size. Panels (a)-(b) fat free mass (FFM), (c)-(d) fat mass (FM) and (e)-(f) body fat percentage (BF%) measured via air displacement plethysmography. Population was divided into two sex-stratified halves, with the first half used to fit male and female-specific linear estimation models and an equally-sized subset containing an even distribution of sexes used to fit the combined sex model. The second half or test set was then randomly and repeatedly restricted with root mean squared error (RMSE) and R-squared determined for each iteration.

To characterise model performance, we evaluated RMSE and R-squared statistics for both sex-specific and combined sex models for a range of test sample sizes, displayed in Fig 2. The R-squared statistics for combined sex and sex-specific models were similar, as also reflected by model fit to the complete dataset in Table 3 (R-squared combined: 0.590, male: 0.589, female: 0.591), though sex-specific models tended to exhibit a lesser RMSE in the estimation of FM, FFM and BF%.

## Discussion

### Summary of findings

In this study, we developed and evaluated a range of models for characterising neonatal nutritional status. Using a composite neonatal morbidity, we developed a model to detect undernourished newborns which exhibited an AUC of 0.740 (0.63, 0.85). We also examined the greatest AUC achieved by a model excluding length as a feature and found that a combination of birthweight, abdominal, head and mid-thigh circumferences yielded a AUC of 0.736 (0.62, 0.85).

Models for identifying low and high BF% neonates exhibited AUCs of 0.827 (0.78, 0.88), and 0.834 (0.79, 0.88), respectively. This suggests potential for application in LMICs, offering a low-cost and scalable approach for screening at birth, though this may depend on measurement accuracy and reproducibility, availability of appropriate equipment, training and evaluation of competency [24]. These factors considered, the models could nevertheless motivate the routine collection of anthropometric measurements, especially considering the socio-economic transition that many LMICs are undergoing, with both under and overnutrition present at birth.

### Neonatal morbidity screening

The combination of birthweight, length, chest and mid-thigh circumferences exhibited the greatest AUC of 0.740 (0.63, 0.85) to identify neonatal morbidity. AUC from leave-one-out cross-validation was 0.698. It was interesting to note the presence of the birthweight-to-length ratio, possibly corrected for chest and mid-thigh circumferences as a potential marker for composite neonatal morbidity. Neither of these circumferences have been routinely used as a marker of adiposity, though chest circumference has been identified as a strong predictor of low birthweight [10] and there have been similar correlations reported between mid-thigh circumference, antenatal nutrition [29] and birthweight [30].

The model excluding length as a feature exhibited a AUC of 0.736 (0.62, 0.85) and did not exhibit a significantly poorer performance than that where length was included. Given the more expensive and bulkier nature of length boards compared with paper tape measures for circumference measurements, this length-free composite measure may be preferred for use in LMICs.

MUAC is a simple and fast measurement widely-used to detect undernutrition in LMICs. Though MUAC in infants under 6 months may have predictive value for infant death [31], the difference between the ROC curves for MUAC and BF% would suggest that prediction of morbidity as defined by our composite measure in this population may be improved by accurate measurement of BF% if available, or by using our anthropometric model, subject to further evaluation and validation in an independent dataset. Our model exhibited an AUC of 0.740 (0.63, 0.85) which was the next highest to BF%, among other comparisons including W/L^2^ (0.726 (0.61, 0.84)), birthweight percentiles (0.695 (0.57, 0.82)) and MUAC (0.655 (0.51, 0.80)) (Fig 1, Table 2).

### Screening of low and high BF% neonates

Both models yielding the greatest AUC for screening low and high BF% contained a ratio between weight and length in some form, with the low BF% neonates consisting of weight, length and chest circumference and the high BF% containing birthweight, weight, length and head circumference.

The developed model for screening low BF% neonates exhibited a AUC of 0.827 (0.78, 0.88), greater than that of the W/L for gestational age model, though the difference was not statistically-significant. In contrast, the W/L for gestational age exhibited the greatest AUC of 0.836 (0.79, 0.88) for identifying high BF% neonates, consistent with previous reports of increasing BF% with increasing gestational age [32], due to rapid fat gain late in gestation [33]. This is followed by both the composite feature and the birthweight percentile models with AUCs of 0.834 (0.79, 0.88), suggesting that for this cohort, accounting for multiple anthropometric measures performs no better than considering the birthweight percentile alone, despite the latter being considered a limited predictor of morbidity and mortality. Such percentiles are nevertheless problematic as gestational age is frequently unreliable in LMICs.

### Linear estimation of neonatal fat

Anthropometric models for the estimation of neonatal fat mass developed by Catalano et al. [16], Schmelzle et al. [20] and Deierlein et al. [18] exhibit an R-squared of 0.84, 0.94 and 0.81, respectively. A direct comparison between our models and those developed previously is difficult given the lack of corresponding skinfold thickness measurements, differences in the criterion method for FM estimation and demographic variations. Our developed models exhibited an R-squared of 0.59 for both male and female populations, accounting for a lower variance in FM than previous reported models. This highlights the important contribution and correlation of skinfold measurements to FM estimation, though without considerable practice, these measures may have poor reproducibility amongst multiple users [34].

Comparisons of sex-specific anthropometry revealed that males tended to be longer and heavier than females, with larger head and chest circumferences on day of measurement (Table 1). The male neonates in our cohort also had lower BF%, which also remains consistent with previous studies [32]. In the anthropometric models previously developed for estimation of body composition [16–20], sex is often not included or adjusted for using a binary variable in the linear regression model. This adjustment for sex differences in anthropometry and body composition may well be inadequate, especially for robust model development. Though variability in body composition measures were similar across both sex-specific and combined sex models, we observed a greater estimation error for the combined sex models (Fig 2) and a different combination of variables that were significantly predictive of fat mass between male and female-specific models (Table 3).

The population-specific nature of body composition also extends beyond sex; there are also differences between infants of different ethnicities [35, 36], that may be genetic, biological, environmental or composites of these. Body composition is also influenced by perinatal characteristics, infant feeding methods [37] and days after birth, with infants undergoing an initial weight loss particularly during the first four postnatal days [28]. In this period, energy intake is limited until breastfeeding is established by day 5 when fat stores are no longer needed as alternative energy stores and energy is expended to the requirements of extrauterine life including thermoregulation, fluid balance and respiration [38, 39]. These factors contribute to high variability observed in the neonatal period and thus need to be considered in robust model development and application.

The changes in RMSE and R-squared with testing set size in Fig 2 demonstrate the influences of both training and testing sets on the performance and robustness of the final model. These results support sex-specific model fitting, highlighting the potentially inflated error from combining both male and female subjects in the same model with a single variable adjusting for sex. It also aids in establishing a context for model evaluation, with different target variables (fat-free mass, FM and BF%) tending towards different degrees of correlation with anthropometric variables. An understanding of model fit and robustness extends beyond R-squared which characterise the relationship between two variables, rather than agreement or differences between them. Despite similar R-squared statistics across both combined sex and specific model estimations of neonatal body composition, the RMSE tended to be lower for sex-specific models.

### Strengths and limitations

The strengths of this study include the large sample size of neonates and the use of ADP as the criterion method which has been specifically validated in this population [13–15]. The model development for detecting neonatal morbidity was limited by the low representation (3.4%) of neonatal morbidity [12]. Although we approached this by using leave-one-out cross-validation for model evaluation, there is a need for further evaluation on a population with higher neonatal morbidity. The optimal measurement of morbidity may change in a different population, as the composite score used for this analysis was based on a logistic regression analysis of potentially significant factors in the given population [12]. This dataset would ideally be sourced from LMICs where demographic characteristics align with the intended area of application for the model.

Due to the accurate measurement of weight using the integrated PEA POD scale, further validation of accurately measuring weight for the models may be required. Anthropometry including length and circumference measurements were also not obtained in duplicate except for a small subset of approximately 40 infants [24].

Body composition estimation in the neonatal population is especially difficult given the varying criterion methods, the growing evidence to suggest poor agreement between gold standards in this population [40] and the variation in body composition during the first few days of life. The model development and potentially, predictive ability, may be improved by adjusting for additional features such as skinfold measurements, which were not collected in this dataset. Evaluation in an independent population to gauge estimation error and robustness of predictive ability for neonatal morbidity, low and high BF% infants is also needed.

## Conclusions

The greatest burden of neonatal and infant undernutrition and morbidity lies in LMICs, where there is an urgent need for suitable, simple approaches to monitor and manage infants. Using combinations of anthropometric features, we fitted models for application in these settings that could detect composite morbidity with an AUC of 0.740 (0.63, 0.85) in neonates in the first few days of life. Composite features involving simple, accurate, easily-measured anthropometric features could also identify low BF% infants with an AUC of 0.827 (0.78, 0.88). These models have demonstrated potential for further development and evaluation in LMICs for identifying infants in need of further nutritional management.

## Supporting information

S1 Dataset Neonatal anthropometric data. (CSV)
